# Korsgaard’s Constitutivism and the Possibility of Bad Action

**DOI:** 10.1007/s10677-017-9851-9

**Published:** 2017-11-28

**Authors:** Herlinde Pauer-Studer

**Affiliations:** Department of Philosophy, University of Vienna, Universitätsstraße 7/3, 1010 Vienna, Austria

**Keywords:** Constitutivism, The categorical imperative (as a constitutive rule and a regulative rule), Justification of the categorical imperative, Bad action

## Abstract

Neo-Kantian accounts which try to ground morality in the necessary requirements of agency face the problem of “bad action”. The most prominent example is Christine Korsgaard’s version of constitutivism that considers the categorical imperative to be indispensable for an agent’s self-constitution. In my paper I will argue that a constitutive account can solve the problem of bad action by applying the distinction between constitutive and regulative rules to the categorical imperative. The result is that an autonomous agent can violate the categorical imperative in so far as it amounts to a regulative rule of morality; however, an agent cannot call into question the categorical imperative as a constitutive rule of the practice of morality without losing her or his identity as a moral agent. The paper then compares this approach to bad action with the one Korsgaard provides and outlines also a new way of grounding the categorical imperative.

Kant’s way of defining the relationship between autonomy and morality seems to make it impossible to ascribe immoral actions to the will and volitions of an autonomous agent. His assumption that an autonomous will is identical with a morally good will restricts freedom of the will to morally good actions.

The problem also comes up in those Neo-Kantian accounts that try to ground morality in the necessary requirements of agency. Christine Korsgaard’s work on self-constitution is the most prominent example. For Korsgaard, the categorical imperative amounts to a constitutive principle of autonomous agency. Again, the question arises: What about bad and evil agents?^1^

This paper argues that a Kantian constitutive account of agency and morality can make space for bad action by applying the distinction between constitutive and regulative rules to the categorical imperative. Reading the various formulas of the categorical imperative in light of the distinction between a constitutive rule and a regulative rule offers the following solution to the bad-action-problem: acting badly often is a violation of a regulative rule, but not necessarily a violation of a constitutive rule of morality. A violation of the categorical imperative, as far as it features as a regulative rule, thus amounts to a failure that does not jeopardize one’s being an agent who, in principle, acknowledges the normative force of the moral law. A violation of the categorical imperative in its constitutive form is a much more serious matter: it might even result in an agent’s loss of moral identity. The position defended here deviates from Korsgaard’s account in the following respect: the categorical imperative is seen as a constitutive principle of the practice of morality, and, thus of moral agency, but not of agency as such.

The paper is structured in the following way. Section 1 outlines Korsgaard’s constitutive account of agency and morality. Section 2 argues that Korsgaard’s attempt to solve the problem of bad action conflicts with her attempt of justifying the categorical imperative. In Section 3, I will present my reading of the categorical imperative, showing in detail in which way the categorical imperative is best understood as a constitutive rule and in which way as a regulative rule. This interpretation suggests a way of grounding the categorical imperative that avoids the bad action antinomy. Section 4 compares this interpretation with Korsgaard’s understanding of the categorical imperative. Finally (Section 5), I try to outline how the account defended here can accommodate bad action.

## Korsgaard’s Account of Self-Constitution and Autonomy

In her book *Self-Constitution: Agency, Identity, and Integrity* Korsgaard claims that “the source of normativity lies in the human project of self-constitution” (Korsgaard 2009a, p. 4). Self-constitution, so her thesis, depends on normative principles, including basic principles of morality. The categorical imperative (CI) thus amounts to a constitutive standard of agency, more specifically: the CI is indispensable for being a unified agent. Let us look at the steps leading to that result.

Constitutive standards are, Korsgaard explains, those “normative standards to which a thing’s teleological organization gives rise” (Korsgaard 2009a, p. 28). Her example is the way a house is built. A house needs walls and a roof, but also a certain form. The different parts must be organized in a way so that the house serves its purpose, which is to provide shelter. A house can be built in a shabby way, but for an object to be a house, it needs a minimal structure of roof and walls. A ‘good house’ fulfils its function well; a ‘bad house’ serves its function poorly.

Something similar holds for agency. Korsgaard’s argument in short: To be an agent, one must be constituted and guided by the principles that make agency possible. These are the principles of practical reasoning, the hypothetical imperative and the categorical imperative. Those principles enable us to deliberate and decide which incentives we take to be reasons for action.

Korsgaard draws an analogy between the principles of logic governing our theoretical thinking and the principles of practical reason. Someone who outright rejects basic logical principles such as, for example, modus ponens is not capable of drawing certain conclusions from certain premises. So a lack in logical thinking turns the mind of a person into a “disunified jumble of unrelated atomistic beliefs, unable to function as a mind at all” (Korsgaard 2009a, p. 67). Similarly, the principles of practical reason, the hypothetical and the categorical imperative, cannot be outright rejected.

According to Korsgaard, action is a form of self-determination: you determine yourself to be the cause of something. In addition, she claims, there must be *a part of yourself* which is separate from the incentive and which chooses on which incentive to act. The hypothetical and the categorical imperative highlight these two different aspects of action. The hypothetical imperative is involved in setting *yourself to be a cause*. Following the categorical imperative ensures that “the cause is *yourself*” (Korsgaard 2009a, p. 72). The hypothetical imperative and the categorical imperative tell us how to will an action, namely by constituting ourselves as the causes of certain ends.

Constitutive standards have a special normative standing: they meet “skeptical challenges to their authority with ease” (Korsgaard 2009a, p. 29). It simply does not make sense to draw them into question. Just as one cannot ignore the constitutive standards of a house in case one is engaged in building a house, one cannot ignore the constitutive standards of agency if one wants to count as an agent. Since self-constitution is inescapable for human beings, the constitutive standards of agency are therefore “unconditionally binding” (Korsgaard 2009a, p. 32).

Korsgaard ties her account of agency to the categorical imperative in its Universal-Law-formulation (FUL). The relevant passage reads:

“When you deliberate, when you determine your own causality, it is as if there is something over and above all of your incentives, something which is *you*, and which chooses which incentive to act on. So when you determine your own causality you must operate as a whole, as something over and above your parts, when you do so. And in order to do this, Kant believes, you must will your maxims as universal laws” (Korsgaard 2009a, p. 72).

The last step in this argument certainly is in need of justification. Why does agency as the capacity to make choices involve a commitment to the categorical imperative in the FUL-formulation? Korsgaard supports this move by what she calls “‘the argument against particularistic willing’” (Korsgaard 2009a, p. 72). ‘Particularistic willing’ means acting on a reason that only would apply to a particular case, without any implication for other cases. Korsgaard considers such a form of willing to be impossible.^2^

Her argument then proceeds from the unfeasibility of particularistic willing to the categorical imperative (FUL) in the following way: If particularistic willing is impossible, willing must be universal; i.e. the maxim of the action must be willed as a universal law. Thus, the categorical imperative is constitutive of willing and agency.^3^

## The Challenge of Bad Action

Kant’s argument, fleshing out autonomy in terms of the categorical imperative, is embedded in Korsgaard’s explanation of self-constitution. This is why her account, not other than Kant’s moral theory, faces the problem of bad and evil action.

The challenge arises in two ways: First, if the categorical imperative amounts to a constitutive principle of agency, then treating others in a morally bad or evil way seems to undermine one’s agency. Second, by tying autonomy to the categorical imperative, it remains a puzzle how one can autonomously (i.e. intentionally and wilfully) choose acting badly.

Korsgaard addresses both problems. Her answer to the first worry is that bad action is a defective form of agency. The goodness or badness of actions depends, she argues, on their contribution to the unification of agency. Good actions constitute you well, bad actions don’t. Bad actions “fail to constitute their agents as the unified authors of their actions” (Korsgaard 2009a, p. 32). Yet, turning yourself into a unified agent “admits of degrees” (Korsgaard 2009a, p. 25). Acting badly thus amounts to a deficient form of self-constitution, but it is still a form of agency.

The problem with that answer is that it provides us with a strange notion of bad and evil action. Doing the bad often comes with careful reflection on strategies and plans how to realize the envisaged bad or evil ends. Agents who are committed to bad and evil actions might still be well-constituted unified agents, i.e. agents who, to phrase it in Korsgaard’s words, “operate as a whole, as something over and above” their parts.

Korsgaard’s reply to the second worry (concerning the tight connection between autonomy and the categorical imperative) is that the will can either display a poor form of autonomy or can adopt the wrong law. In a case of defective autonomy, the agent choses voluntarily the will of someone else as the governing principle of her or his will.^4^ To illustrate how the will might adopt the wrong law, Korsgaard refers to Plato’s discussion of defective constitutions and defectively governed souls in the *Republic*. Just as a city or society can be governed by the wrong law, so the person can be governed by a law that is formally a law, but substantively unjust or wrong: in Plato’s case a principle of honor (timocracy), or of prudence (oligarchy), or wantonness (democracy) or obsession (tyranny) (Korsgaard 2009a, pp. 163–170).

This suggestion seems, however, incompatible with Korsgaard’s justification of morality. In grounding the categorical imperative she follows Kant’s strategy in the *Groundwork* that works from autonomy of the will to the Formula of Universal Law (FUL) and from there to the Formula of Humanity (FH). Spelling out the meaning of autonomy in terms of the categorical imperative provides us, she argues, “with a bridge into moral territory” (Korsgaard 2007, p. 20).

To outline her argument in more detail: A free will or an autonomous will acts according to its own principle or norm, that is to say, it is guided by a self-given law. The principle of a free will is henceforth a law, and the feature of ‘being a law’ is exactly fulfilled by the categorical imperative in the Universal Law formulation (FUL). The categorical imperative in this version meets the essential property of a law, namely its universality.^5^

This argument establishes a necessary connection between autonomy and the categorical imperative: an autonomous will is a moral will.^6^ But when it comes to explaining bad action, autonomy is not defined in terms of the categorical imperative. Korsgaard’s depiction of bad action as an instance of choosing the wrong law presupposes that there are laws other than the categorical imperative. Yet, if the categorical imperative is the constitutive principle of the autonomous self, then an autonomous person cannot choose a wrong or evil law as her constitutive principle.

The upshot is: In order to make room for bad action, the thesis that autonomy necessarily commits an agent to the categorical imperative must be dropped. But giving up the assumption that an autonomous will is identical with a moral will entails that the justification of morality cannot rely on this assumption. We need a different strategy to ground morality.

## The Categorical Imperative as a Constitutive and Regulative Rule

In this section, I will develop a reading of the categorical imperative in terms of the distinction between constitutive and regulative rules that, as I then try to show, allows for a solution of the bad-action-problem by way of an alternative justification of the categorical imperative.

In the philosophical literature, the concepts ‘constitutive rule’ and ‘regulative rule’ have commonly been explained by their role in practices. Constitutive rules define and make up a practice or, as some authors claim, an institutional fact (Searle 1995, pp. 43–51; Rawls 1955/1999, pp. 36–40). Regulative rules tell those who partake in the practice what to do: their function is to guide. One way to mark the distinction between the two kinds of rules is by the degree of error, in other words the kind of challenge, they allow. A constitutive rule cannot be outright dismissed. Rejecting the constitutive rules of a practice means losing one’s status as an agent who is engaged in that practice. A violation of the regulative rules of a practice, however, amounts merely to a deviation from the rule; the rule as such is not called into question.^7^

Games illustrate the working of constitutive and regulative rules. Take chess. In this case, the very rules that make up the game equally guide the players. And there is a clear line with respect to the violation of the rules. If I move my pawn erroneously crosswise, my partner will correct me. Committing such a mistake does not endanger my playing chess with my partner. But if I move my pawn repeatedly crosswise, my partner might stop at some point, telling me that I obviously gave up playing chess with him. The same holds for other games. If, for example, a basketball player goes wild and ignores the rules, the referee will eventually exclude him. The player’s actions are interpreted as his not willing to participate in the game. To express the point in terms of professional status or identity: A basketball player may in some instances violate the regulative rules of the game; but he cannot challenge the constitutive rules of the practice without losing his status (identity) as a basketball-player. Equally, a chess-player is committed to the rules of chess; she cannot change them at will. The identities of a basketball-player or a chess-player depend on their acceptance of the rules. Applied to the issue of agency and morality we get: an autonomous agent can violate the regulative rules of morality. However, an agent cannot outright reject the constitutive rules of the practice of morality without eventually putting his identity as a *moral* agent at risk.

Games show that constitutive rules are best explained with respect to their functional role in making up *practices*. The analogy with games entails that we first look at the constitutive rules of morality as a practice, and from there we then move on to explain the individual’s status as moral agent. We define moral agency and moral identity in terms of partaking in the practice of morality.

Note that the focus on practices is not just due to a comparison of morality with games. There is a systematic point for taking that perspective. Morality is best understood in a relational way, namely as a matter of making claims on each other and assessing the validity and rightfulness of those claims. Moreover, reflecting on the constitutive conditions of morality, understood as a social practice, helps us to a justification of the categorical imperative that does not rely on grounding the categorical imperative in agency as such.

Let us now turn to the interpretation of the categorical imperative in light of the distinction between regulative and constitutive rules. Kant offers us five formulas of the categorical imperative, three main formulas (the Formula of Universal Law, the Formula of Humanity, the Formula of Autonomy) and two variants, namely the Formula of the Law of Nature (which is a variant of the Formula of Universal Law) and the Formula of the Realm of Ends (as a variant of the Formula of Autonomy).^8^ Kantian philosophers disagree as to which of those formulations they consider to be paramount, but usually either the Formula of Universal Law (FUL) or the Formula of Humanity (FH) or the Formula of Autonomy are singled out as the overriding principle of morality.^9^ However, given our understanding of morality as a social practice, the Formula of a Realm of Ends (FRE) – the requirement to “act in accordance with the maxims of a member giving universal laws for a merely possible realm of ends” (Kant 1785/1996: p. 88; G 4:439) – becomes pivotal.^10^

Kant describes the idea of a realm of ends as “a systematic union of various rational beings through common laws” (Kant 1785/1996, p. 83; G 4:433). As Kant explains, the realm of ends takes shape once we “abstract from the personal differences of rational beings as well as from all the content of their private ends” so that we are “able to think of a whole of all ends in systematic connection (a whole both of rational beings as ends in themselves and of the ends of his own that each may set himself)” (Kant 1785/1996, p. 83; G 4:433). What Kant means is that members of a realm of ends must consider whether their maxims involve ends that are – in principle – compatible with the ends of others. This entails not pursuing maxims involving ends where the corresponding maxims must be reasonably rejected by others.^11^ In other words, FRE demands pursuing ends for reasons that rational agents can share. Meeting this requirement results in a community of rational beings whose relations to each other are regulated by common laws.

The suggestion to consider FRE as central is supported by some passages of the *Groundwork,* though, admittedly, Kant himself did not work out this option fully. According to Kant, FRE provides the complete form of moral reflection by including the moral ideas emphasized by the other formulas of the categorical imperative, namely universal lawgiving (unity of the will), the kind of ends (set by the restriction of treating human beings as ends in themselves), and the possibility of shared ends (system of ends). And he adds that “*a complete determination* of all maxims” is achieved by the Formula of a Realm of Ends, that is, the requirement “that all maxims from one’s own lawgiving are to harmonize with a possible realm of ends as with a realm of nature” (Kant 1785/1996, p. 86; G 4:436).

Shifting the main focus on FRE suggests, I think, seeing the different formulas of the categorical imperative as being related to each other in the following way: FRE is fundamental and the other formulas of the categorical imperative are particular ways of spelling out the normative elements embraced by FRE in terms of criteria for evaluating our maxims.^12^ How should we flesh that out in terms of regulative and constitutive rules?

The regulative rule character of the categorical imperative is apparent in the testing procedures. An important part of Kant’s ethics consists in a procedural assessment of the moral permissibility or impermissibility of one’s subjective principles of action, one’s maxims. The regulative element in the Formula of a Realm of Ends (FRE) is the requirement that all maxims are “to harmonize with a possible realm of ends” which means that we have to ask ourselves whether our maxims can function as common laws. Other than in the case of FUL and FH, Kant does not illustrate the working of this directive by examples. But it obviously helps moral deliberation to ask whether our subjective principles involve claims on others that can or cannot pass the test of mutual recognition.^13^ Moreover, it makes sense to interpret FUL and FH as the outcome of translating the regulative requirement that maxims can function as commonly accepted rules of the moral community into specific procedures for evaluating our maxims.

The test associated with the FUL formula of the categorical imperative is whether one can universalize one’s maxim of action without running into incoherence. The testing procedure spells out this requirement in two versions, namely whether the universalization yields a contradiction in conception or a contradiction in willing. In the first case, we cannot even think of the universalization of the maxim without facing a kind of conceptual incompatibility; the contradiction in willing means that though we can think a maxim to hold universally, we ourselves would not rationally will to live in a social world in which such a rule holds. Kant equally reads the Formula of Humanity (FH) as providing a test for the moral permissibility or impermissibility of maxims. The requirement to treat others as ends and not merely as means, as expressed by FH, is directed at ruling out those maxims that reduce humans to mere instruments used by others for pursuing their interests. Kant’s test is whether I could assent to another person’s mode of acting towards my own person (given that the other person’s maxim would be revealed to me).

The details of Kant’s testing procedures, whether they work and accomplish their purpose, are not crucial for our discussion.^14^ The important point for my argument is that the categorical imperative can be understood as a rule that guides us, i.e. that it gives rise to an assessment of maxims that has normative impact on our ways of acting.

What about reading the categorical imperative in the constitutive mode? We need to distinguish between two senses of ‘constitutive’. In one sense, it means to ask for the regulative rules that make up the ‘game’. As the case of games illustrates, a regulative rule might also be a constitutive rule. This equally holds for the regulative rule-versions of the categorical imperative. Why? Because it seems part and parcel of morality and moral deliberation to ask whether we commit ourselves to maxims that pass the tests of acceptability by others (FRE) and thus the tests of universality (FUL) and respect for others (FH). In this sense, the regulative rule functions also as a constitutive *rule* of morality. In a second sense, however, asking for the constitutive rules of morality means asking for the constitutive *conditions* that are indispensable for the practice. The difference is: while the notion of ‘rule’ has an immediate regulative connotation, the notion of ‘constitutive *condition*’ seems more apt to highlight the indispensability of normative elements, i.e. that they cannot reasonably be called into doubt if one is engaging in the practice.^15^ Constitutive conditions in this sense define a *specific* practice. Thus, reflection on the constitutive role of the categorical imperative means to look for that formula of the categorical imperative in which all indispensable conditions of morality find expression.

Obviously, the first two main formulas of the categorical imperative, the formula of Universal Law (FUL) and the Formula of Humanity (FH), involve normative ideas that are crucial for morality, namely universality and respect for others as persons. However, the way in which Kant formulates FUL and FH, namely as offering guidance for the first-personal assessment of one’s maxims and hence as regulative rules, does not stress the meaning of universality and equal respect as indispensable conditions of the practice of morality. It is in connection with the idea of a realm of ends that the constitutive role of those normative concepts is highlighted. As I have already pointed out, the idea of a realm of ends (which stands for the practice of morality) contains all normative elements that come up in the various formulas of the categorical imperative, namely universality (universal lawgiving), respect for persons (treating others as an end in itself), and acceptability from the standpoint of all those affected by a regulation (common laws).

It is time to come back to the main argument of this paper. In order to sidestep the bad action-antinomy we need to come up with a form of grounding the categorical imperative that does not rely on the analytic identification of an autonomous will with a moral will. My claim is that we find the resources for such an alternative justification in focusing on the normative reasons we have for consenting to the constitutive conditions of the realm of ends.

Let us start with a look at Kant’s methodological approach. Kant seeks to justify the categorical imperative by a transcendental argument. The structure of such an argument consists of a two-step procedure: first, to lay bare the conditions for the possibility of X (knowledge, discourse, an institution, a practice, just to name a few examples), and then, in a second step, to provide normative reasons for the reflective endorsement of those conditions. Such ‘conditions for the possibility of’ are, I suggest, nothing other than presuppositions that amount to constitutive conditions. Their reflective approval completes a transcendental argument.^16^

Kant’s reasoning in the *Groundwork* leading to FUL and FH follows that line. The first step, namely exposing the categorical imperative as a condition for the possibility of moral reasoning and, thus, of acting morally, is performed by what Kant calls a ‘regressive argument’. FUL, as we know, comes up at the end of an analysis intended to identify the principle underlying the good will. Kant proceeds by exposing the conditions such a principle has to meet. The required principle must have the form of a categorical, not a hypothetical, imperative and must amount to a merely formal law. And the argument ends by stating that those conditions – formality, universality and categorical bindingness – are exactly met by FUL.

Kant’s regressive argument for FH draws on two assumptions: first, that our capacity for setting ends defines us as human beings; second, that the rational capacity for ascribing objective values to ends presupposes that one must ascribe objective and thus unconditional value to oneself and one’s rational willing which amounts to respecting humanity in us. Kant derives FH by a regressive depiction of the conditions that underpin our valuing ourselves as human beings and rational agents. The argument begins by claiming that characteristic of rational beings is the capacity of self-determination by a will. Kant then states that the ground of the will’s self-determination has to be an objective end, not an end that is only conditionally valuable.^17^ And he concludes that all rational and all human beings meet this condition; they can never have merely conditional value and can never serve merely as means. Again, Kant comes up with conditions which the sought principle has to meet, namely: it must hold categorically, it must be “an *objective* principle of the will”, and it must be able to “serve as a universal practical law” (Kant 1785/1996, p. 79; G 4:429) – conditions which are exactly fulfilled by FH.

Kant acknowledges that the regressive arguments do not amount to a full justification of the categorical imperative. This is why he undertakes a “deduction” of the categorical imperative in Section 3 of the *Groundwork*. The deduction proceeds from the claim that a rational will is an autonomous will and that an autonomous will and a moral will are one and the same to the conclusion that a rational will is a moral will. However, Kant himself was uneasy with his argument; he suspected it to be circular, simply presupposing the autonomy of the will without a further argument. That he indeed had every reason to be concerned is evident given his underlying assumption: “If, therefore, freedom of the will is presupposed, morality together with its principle follows from it by mere analysis of its concept” (Kant 1785/1996, p. 95; G 4:447). Kant’s argument relies on an analytic connection between autonomy and morality, and, thus, on exactly that presupposition which gives rise to the bad-action-problem.

The upshot is: Kant’s deduction, which is meant to complete the transcendental argument, is not successful, because it rests on simply postulating that morality follows analytically from autonomy. Kant fails to provide additional reasons why the reflective endorsement of the categorical imperative seems inevitable.

However, there is a further point we need to take into account. Kant’s deduction, even if it were successful, would only show that the categorical imperative is the principle of individual autonomous agency. His justificatory attempt remains within an unduly restricted first-personal paradigm. So, Kant’s own argument does not include what he himself, when he talks about the realm of ends, considers to be a constitutive condition of morality, namely that the laws of morality must be the outcome of *common* legislation. Note: Though I think that there are independent arguments why we should conceive of morality in a relational way, namely as a matter of making claims on each other and being accountable to each other, my objection here is simply that Kant himself, given his own account of the realm of ends, must provide an argument for grounding the corresponding version of the categorical imperative, i.e. FRE. Kant’s regressive arguments expose the constitutive rules of rational willing and autonomy, but not the constitutive conditions of morality as a practice. He makes some steps in that direction (recall his claim that FH gives rise to the idea of a realm of ends), but the argument why the realm of ends is of central importance is not fully developed.

To fill that lacuna by a transcendental argument, we have, first, to lay bare the constitutive conditions of morality as well as the corresponding principle covering and embracing them, and, second, we have to come up with reasons for endorsing that principle.

I think I have already said enough about the regressive exposition of the constitutive conditions of morality (universality, equal respect, and commonality) and why FRE is the principle that unites all those conditions and thus matches the idea of a realm of ends. But what about the second step of the transcendental argument?

In a nutshell, the challenge facing us at this level can be put this way: What considerations can we provide for making the reflective endorsement of FRE compelling? Here is an answer: Forming our social interactions on the model of a realm of ends grants us the status of being agents who relate to each other in terms of respect. Thus, we all have normative reason^18^ to reflectively approve of FRE because this principle is constitutive for preserving our dignity. To put it differently: Given that we affect each other by our actions, we need normative regulations enabling us to live together in a cooperative way and entertaining relations of respect and thus accountability to each other. Otherwise we would be in a social “state of nature”, not in a morally “righful condition”.^19^ That insight yields a compelling consideration for endorsing FRE because there is no reasonable practical point of view from which it could be rejected.^20^ This completes the second step of the transcendental argument.

Note that the argument outlined above depicts general normative reasons for endorsing FRE, reasons that rational agents can share. However, the argument also speaks to the individual standpoint: it provides *me* with a normative reason to consent to *my* membership in such a community. Since I live in a world in which the actions of others have an impact on me, I have a normative reason to endorse principles that grant me the normative status of an autonomous agent and obligate others to treat me accordingly. Otherwise, I would be at the disposal of others’ arbitrary wills – degraded to an object, a kind of instrument. To avoid such a condition, I have a reason – note, a normative reason beyond mere instrumental reasoning – to recognize the normative force of the constitutive principle of morality and hence to adopt a moral identity. Autonomous practical reflection thus brings socially vulnerable agents to accept the moral law, and this involves accepting also the regulative rules of the practice, namely the categorical imperative in form of the procedural testing rules of FUL and FH.^21^

The commitment to being part of the moral community constitutes me as a moral agent. Just as the basketballer gains his status and identity by endorsing the rules of basketball, my status as moral agent and thus my moral identity is secured by endorsing, in principle, the basic rules of morality. Note that this entails a willigness on my side to comply with the regulative implications of the rules; however, this compliance still allows for particular instances of violating the rules on the regulative level. Such particular performance errors do not yet endanger my moral identity. What we have phrased in terms of my perspective also holds for *all others*, seen from their individual perspective.

To recapitulate this section: First, I have applied the distinction between constitutive and regulative rules to the various formulas of the categorical imperative. Reading the categorical imperative in those terms has led me to propose FRE as the constitutive principle of the practice of morality and FUL and FH as involving testing procedures that display the categorical imperative as regulative rules. I have then outlined the two steps of justifying FRE by a transcendental argument. And finally, I tried to show in which respect the shared normative reasons for endorsing FRE provide us with individual normative reasons for understanding ourselves as moral agents.

## Korsgaard’s Distinction Between the Moral Law and the Categorical Imperative

How does the account developed in the preceding section fare in comparison with Korsgaard’s understanding of the categorical imperative and, moreover, her distinction between the categorical imperative and the moral law?

Let us first turn to looking at Korsgaard’s analysis of the categorical imperative through the lens of the distinction between regulative and constitutive rules. Recall that the regulative character of the categorical imperative is apparent in the procedures for testing the permissibility or impermissibility of maxims. For Korsgaard, deliberative procedures form the core of Kant’s ethics and, accordingly, she sees the categorical imperative as being tied to such a procedure:

“Morality, on Kant’s account, is not a certain set of considerations, identified by their content, but a *way* of deliberating: the categorical imperative […] is part of the structure or *logic* of practical reason. […] (W)hat distinguishes the moral agent, on Kant’s account, is not first and foremost *what* he thinks about when he decides, but rather *the way* he deliberates when he makes his decisions” (Korsgaard 2009a, p. 48; italics in the original).

Kant, she argues, gives us a “‘testing’ rather than a ‘weighing model’ of reasons” (Korsgaard 2009a, b, p. 51). She discusses in detail the procedural assessments of our maxims to which FUL as well as FH give rise. Korsgaard considers the test associated with the Formula of Humanity, namely whether a person could possibly assent to the maxim of another specifying how that other person intends to act towards her, to be important for elucidating coercion and deception (Korsgaard 1996b, p. 138 f.). And she endorses the universalization test for maxims, as exemplified by FUL, as indispensable for moral deliberation. In fact, formal procedures, as exemplified by FUL, seem to her more reliable than intuitive appeals to substantive moral reasons. Here is why: Substantive moral reasons are, she claims, conditionally valid, dependent upon the acceptance of a particular substantive conception of moral rightness or goodness. In the substantive sense, “a ‘moral reason’ is simply one among many considerations, and its status as unconditional can therefore be called into question” (Korsgaard 2009a, p. 49). A formal account of moral reasoning does better, she argues, because it is based on a correct deliberation procedure and therefore gives rise to reasons that are “unconditionally binding” (Korsgaard 2009a, b, p. 49), not reasons which are dependent on the endorsement of substantive moral values which themselves are in need of justification. In the background here is her approval of constructivism, according to which a procedure alerts us, first, to identify the reasons that are morally relevant and then, secondly, to assess the reasons in light of a testing procedure. All this shows clearly that Korsgaard endorses the categorical imperative as a regulative rule (though she does not use the term).

What about the categorical imperative as a constitutive rule? Korsgaard’s aim is, as we have seen, to establish the categorical imperative as a constitutive principle of agency. The relevant point for us, however, is: Does she ever present the categorical imperative as a constitutive principle of morality? Korsgaard indeed comes close to doing so when she introduces the moral law in *The Sources of Normativity*. Her explanation shows that she conceives of the moral law as the constitutive principle of a community akin to a realm of ends. As Korsgaard writes:

“Now I’m going to make a distinction that Kant doesn’t make. I am going to call the law of acting only on maxims you can will to be laws ‘the categorical imperative’. And I am going to distinguish it from what I will call the ‘moral law’. The moral law, in the Kantian system, is the law of what Kant calls the Kingdom of Ends, the republic of all rational beings. The moral law tells us to act only on maxims that all rational beings could agree to act on together in a workable cooperative system” (Korsgaard 1996a, pp. 98-99).

Korsgaard’s separation between the moral law and the ethical categorical imperatives makes sense in so far as one reads the moral law as an application of the categorical imperative to morality as a social practice. Seen this way, the moral law amounts to a constitutive rule of morality as it is exemplified in the idea of a cooperative system based on principles to which all could consent.^22^ Such an interpretation is confirmed by what she says about the *substantive* mode of the categorical imperative (another term, I think, for the moral law) in her later book. In *Self-Constitution* she characterizes “the categorical imperative in the more substantive sense necessary for morality” as “a universal law that governs all rational beings, yielding reasons that all of us can share” (Korsgaard 2009a, 181). In this mode, the categorical imperative amounts to a grounding principle of morality.

So far, we have shown that Korsgaard’s discussion of the moral law and the categorical imperative squares with the suggested reading of the categorical imperative as a constitutive rule as well as a regulative rule. In fact, taking into account this distinction helps in clarifying her position.

What about the claim of this paper to take the realm-of-ends-formula of the categorical imperative as central? When we compare Korsgaard’s formulation of the moral law with FRE, we hardly see a difference. Her characterization of the moral law as “the law of what Kant calls the Kingdom of Ends, the republic of all rational beings” shows that the moral law corresponds to FRE, the formula of the categorical imperative that is tied to the idea of a realm of ends. And her explanation that “(t)he moral law tells us to act only on maxims that all rational beings could agree to act on together in a workable cooperative system” not only expresses what FRE requires, namely to act “in accordance with maxims of a universally legislative member for a merely possible realm of ends”, but also emphasizes the aspect of commonality involved in the idea of a realm of ends. Given the importance Korsgaard attributes to the moral law, it seems safe to conclude that her account concurs with the interpretation I offered in the preceding section.

However, there remains a crucial difference. Other than the account defended in this paper, Korsgaard does not provide a grounding of the moral law that corresponds to understanding it as the constitutive principle of morality, something necessary for making plausible the importance she attributes to that principle. Though Korsgaard’s explication of the meaning of the moral law comes close to defending a relational account of morality, her justification of the moral law does not match that move. Korsgaard still seeks to ground the moral law by presenting it as the constitutive principle of autonomous individual agency. This is evident in the *Sources of Normativity* where she concedes that her argument merely proves that “*the categorical imperative* is the law of a free will. But it does not establish that the *moral law* is the law of a free will” (Korsgaard 1996a, p. 99; italics in the original).

Korsgaard follows, as already mentioned, Kant’s justification of the categorical imperative in the *Groundwork*. Kant reasons, as she points out, from the assumption that “because the will is a kind of causality, it must operate according to a law” to the “conclusion that ‘a free will and a will under moral laws are one and the same’” (Korsgaard 2009a, p. 79; referring here directly to Kant’s *Groundwork*, 1785/1996, p. 95; G 4:447). She just thinks that with respect to justifying the moral law, this argument needs to be supplemented in two respects: first, we must assume that the moral law ranges over all rational beings, and second, that the reasons involved in universal maxims are public and shareable. Her point is simply that the individual moral agent is *qua* agent committed to universal maxims and that “the reasons embodied in universal maxims must be understood as public” and thus must “have force for all rational beings” (Korsgaard 2009a, p. 80).

But those additions do not establish the moral law as the constitutive principle of morality – morality conceived as “a workable cooperative system”. The domain requirement merely states that the moral law is binding for each rational agent and thus, aggregatively, for all rational agents: in the same way that it has normative force for me, it has normative force for each other agent. But that is a relation between the moral law and the individual rational will, considered in isolation. The publicity condition maintains that reasons are not reduced to what Korsgaard calls “private mental entities” (Korsgaard 1996a, p. 131). Yet, the publicity condition is already fulfilled if I can communicate *my* reasons for action to others. But those reasons might be reasons that only speak to my perspective, for example my egoistic interests, and might not be approvable by others, given their perspective. As R. Jay Wallace puts it: “The movements of the will that make principles normative for me do not automatically determine what you have reason to do” (Wallace 2009, p. 493).

Actually, in the *Sources of Normativity*, Korsgaard tries to use the publicity argument as a route to a kind of relational account of reasons. She connects ‘publicity’ to shareability by arguing that the “public character of reasons is indeed created by the reciprocal exchange, the sharing, of the reasons of individuals” (Korsgaard 1996a, p. 135). What she means here is obviously not the mere public exchange of reasons, but shareability in the sense of a common agreement on the normative force of reasons. In this stronger sense, reasons are shared when we recognize and acknowledge that there is no reasonable vantage point from which their normative impact and force can be reasonably rejected. However, this common perspective, which is part and parcel of the moral law, requires that the introduction and justification of the moral law is backed not merely by the autonomy of the individual rational will but by an endorsement of all. Korsgaard’s emphasis of shareability is in tension with her locating the source of normativity solely in the self-legislation of the agent. Just like Kant she does not adopt the plural subject perspective to which she is committed by emphasizing the moral law.

The move from the individual will and, thus, individual agency to the moral law does not yield the kind of community that Korsgaard associates with the moral law. She depicts the moral community as “a workable cooperative system”. But her argument merely establishes an association of individually rational and autonomous beings who are universal lawgivers with respect to their own ways of acting. Yet, Kant’s “republic of rational beings”, which inspires her formulation of the moral law, is held together by *common* laws. Korsgaard offers an argument about the possibility of individual moral agency, but not a transcendental argument for morality. She introduces the moral law but does not spell out its relational normative force. Her anchoring the moral law in rational willing, and hence agency, does not yield an argument for why we should commit ourselves to entertaining *cooperative* relations to each other. Korsgaard’s justificatory strategy does not fit with what, given her own interpretation, the moral law requires, namely, shaping our relations to each other on the model of “a workable cooperative system”.

However, my suggestion of reading the moral law as a constitutive condition of the practice of morality meets the features that Kant attributes to the realm of ends. The result is a community that displays a more robust sociality than the mere co-existence of individually rational lawgivers. On the account defended in this paper, others are co-deliberators and thus agents who are trying to achieve an agreement on binding principles for the community.

We need to address a possible criticism. One might object that the outlined way of arguing drives us back to Korsgaard’s step from autonomy to the categorical imperative and thus undermines our claim to offer an alternative justification of the moral law. Let me clarify: My point has been that by committing ourselves to compliance with the moral law we give ourselves a moral identity. Such a commitment, if it is not due to compulsion or mere force, must of course be the outcome of autonomous deliberation and willing. Defining ourselves to be members of the moral community means consenting to its constitutive principles. However, this neither anchors the *justification* of the moral law (categorical imperative) in our autonomy as an agent nor in our moral identity. Our approach, to repeat, pursues a different line for grounding the moral law. Instead of claiming that autonomy of the agent necessarily entails a justification of the moral law, we first ask what principle constitutes a community of human beings who respect each other and shape their relations by common rules. This leads to the moral law (in the form of FRE) and the shared normative reasons for accepting it. And only in a next step the question arises what individual normative reason the agent has to consent to membership in such a community.

This last point gives rise to another possible worry. One might criticize that by rejecting Korsgaard’s attempt of anchoring the categorical imperative in agency, the account offered in this paper runs into the problem of not being able to answer the “why-be-moral”-question. The crucial point of Korsgaard’s program, so the objection goes, is exactly to foreclose this question: being an agent simply rules it out. As a consequence the moral skeptic is silenced.^23^

Now, a central thesis of this paper is exactly that we cannot, and also should not, aim to show that a commitment to morality is absolutely necessary and inevitable. The most we can offer is to provide the normative reasons why such a commitment seems not rejectable from the standpoint of practical rationality (in the sense of reasonable reflection). And here is why we should rest content with that result. It is this space left by my account, i.e. that an agent’s commitment to morality is not a necessary entailment of his being an agent but remains dependent on rational reflection, that makes room for bad action (in the grave sense of a non-commitment to morality).

To summarize this section: First, I tried to show that the suggested reading of the categorical imperative as a constitutive rule as well as a regulative rule squares to a remarkable extent with Korsgaard’s distinction between the moral law and the categorical imperative. Second, I argued that the introduction of the moral law makes Korsgaard’s account similar to the position defended in this paper, namely to consider FRE as the paramount version of the categorical imperative. But I have objected that her justification of the moral law fails to use the normative resources offered by the idea of a realm of ends and still relies on the analytic connection between an autonomous and a moral will. Thus, her approach does not overcome the problem of bad action.

## Bad Action: An Answer to the Challenge

Our attempt to cope with the issue of bad action seems more promising: neither the introduction nor the justification of the moral law (FRE) depend on the assumption that an autonomous will and a moral will are one and the same. We thus are able to make room for what Korsgaard accentuates, namely that evil comes with chosing the wrong law.

The account proposed in this paper not only accomodates bad action, but keeps two main forms of bad action apart. Bad action can be a violation of a regulative rule. But bad action might take a more serious direction: it can be due to the person’s commitment to principles that conflict with the constitutive rule of the practice of morality. And such a commitment to bad and immoral principles might even yield a person’s loss of moral identity.

It seems obvious that an agent can violate the categorical imperative as a regulative rule without undermining his or her agency. An agent might go through the deliberation procedure and still not accept its outcomes. She or he might not be willing to accept the normative obligation coming with the categorical imperative test, namely to drop a maxim that cannot be universalized, and hence she does not carry out the action licensed by that maxim. Equally, an agent might not be willing to give up maxims that do not pass the test of non-instrumentalization of others. Such violations concern the particular case, not the practice of morality as such. Partaking in the practice would be at stake if the agent rejected the constitutive conditions of morality and hence also the associated testing procedures altogether. Drawing the line between constitutive and regulative rules thus helps us to a more nuanced picture of bad action.

Korsgaard refers to the more severe form of bad action when she talks of the person’s choice of the wrong law. Given her framework, which depicts the categorical imperative as a constitutive principle of agency, bad action thus involves a change on the level of self-constitution as an agent. But that seems implausible: mundane forms of acting badly amount to violations of regulative rules. Moreover: agents who violate rules of morality to an extent and in a way that challenges their moral identity are still agents. What is at stake is not their agency per se, but their moral agency in terms of their commitments to morality and hence their individual virtuousness (or badness).

Korsgaard’s account faces another grave problem. Since she ties the normative status of the categorical imperative to the constitution of the agent, a consequence is that bad action would affect not only the normative and moral identity of the person, but also the normative standing of the categorical imperative.

The problem is apparent in Korsgaard’s reply to an objection raised by G.A. Cohen. Cohen picks up on Korsgaard’s claim that the source of normativity lies in the self-legislation and thus identity of the person. How, Cohen asks, can we deny that an ideal Mafioso who is committed to “a code of strength and honour” (Cohen 1996, p. 183) displays autonomy, self-legislation and reflective endorsement? His objection is that the ideal Mafioso who is bound by principles has autonomy “as much as anyone does, this capacity to transcend impulses through reflection and endorse or reject them” (Cohen 1996, p. 184). The ideal Mafioso fulfills, according to Cohen, the criteria of Korsgaard’s argument leading to the categorical imperative – but obviously he is far from meeting that principle.

Korsgaard approves of the similarity between the morally obligated person and the ideal Mafioso: his obligations are not spurious, but, as she argues, real obligations (Korsgaard 1996d, p. 257). What brings the Mafioso to give up his commitment to the Mafia code, she claims, is reflection on the value of his humanity – a value that, for Korsgaard, “stands behind our other roles and imparts normativity to them” (Korsgaard 1996d, p. 256). By reflective thinking, which is inevitable for human beings, the Mafioso realizes that he can no longer lead a life based on the Mafia principles. Korsgaard concedes that such a move “depends on how much of the light of reflection is on” (Korsgaard 1996d, p. 257).

But this reply to Cohen does not solve the crucial issue, namely that the normative standing of the categorical imperative cannot rise and fall with the reflective capacity of the Mafioso (a character who stands for any morally deficient agent). What the Mafioso’s reflection controls and influences is his own commitment to the principles of morality. But the normative force of those principles must hold independently from the individual’s compliance with those principles.

The account I offer avoids this problem because it presents a justification of the moral law (which is the constitutive principle of morality) that is independent from the individual agent’s commitment to this principle. Reflection might bring the individual agent to recognize that she has a normative reason to endorse the constitutive rule of the practice of morality: this will grant her the respect of others and thus help her to a self-understanding in accordance with valuing her own humanity. The agent’s normative identity is shaped by her or his acceptance or dismissal of the categorical imperative; but the validity of the categorical imperative as the grounding principle of morality holds even if an agent falls away from it.^24^

## Conclusion

Korsgaard’s detailed exposition of a constitutive account of the self is a philosophically impressive and eminent contribution to our understanding of the connection between agency, self-understanding and morality.^25^ Korsgaard, one might add, aims to work out a Kantian idea about which Henry Sidgwick had the following to say: “(N)othing in Kant’s ethical writing is more fascinating than the idea – which he expresses repeatedly in various forms – that a man realises the aim of his true self when he obeys the moral law” (Sidgwick 1907/1962, p. 516). This paper has tried to show that by taking into account the difference between constitutive and regulative rules constitutivism might answer the perhaps most crucial problem it faces, namely to explain how one might violate the principles of morality without jeopardizing one’s status as an agent.

## Footnotes

1 Note that in the *Groundwork* Kant equates an autonomous will with a morally good will. In the *Religion*, Kant distinguishes two forms of free volition, namely *Wille* (the autonomous or free will based on the moral law) and *Willkür* (the free power of choice). The distinction between the free power of choice and the autonomous will is crucial for Kant’s account of evil, particularly radical evil, in the *Religion*. Radical evil consists, Kant claims, in an inversion of the moral order, i.e. the subordination of the moral law to the law of self-love (which clearly presupposes freedom of choice). See Kant (1793/1998, pp. 45–61; R 6:6.20–6.38). Some philosophers (Allison 1990, ch. 7 and ch. 8; Sussman 2005) assume that by introducing the distinction between *Wille* and *Willkür* Kant has solved the problem of bad action. However, the problem remains as long as the justification of the categorical imperative relies on the identification of an autonomous will with a morally good will. Kant’s *Religion* is here cited by the page numbers of the CUP edition and R as an abbreviation of the title together with the location of the passage in the Akademie edition of *Kant’s Gesammelte Schriften*. References to Kant’s *Groundwork* (abbreviated with G) are to the edition of the text in *Practical Philosophy*, translated and edited by Mary Gregor (together with the page numbers in *Practical Philosophy* and the location of the passage in the Akademie edition). With one exception (“realm of ends” instead of “kingdom of ends”) the translations of Kant’s formulations are taken from Mary Gregor’s edition of the text in *Practical Philosophy*.

2 I take her argument establishing this conclusion to be: 1. Willing requires identification with the principle of choice on which you act. 2. Particularistic willing has no principle of choice except the specific particular incentive. 3. A particularistic incentive is reduced to the inclination of the moment, not representing a type or kind. 4. Particularistic willing requires you to identify with the inclinations and thus impulses of the moment. 5. Having a particularistic will thus reduces you to “a series, a *mere* heap, of unrelated impulses” (Korsgaard 2009a, p. 76). 6. Therefore: Particularistic willing is not willing at all. The argument is outlined in Korsgaard 2009a, pp. 72–76. Another way in which Korsgaard expresses her objection is: “A truly particularistic will must embrace the incentive in its full particularity: it, in no way that is further describable, is the law of such a will. But this means that particularistic willing eradicates the distinction between a person and the incentives on which he acts” (Korsgaard 2009a, p. 76).

3 The complete argument leading from action and self-constitution to the categorical imperative in its Universal-Law formulation (FUL) can be reconstructed in the following way: 1. We need to act in order to be alive (who we are is constituted by our choices). 2. Action is a form of self-determination. 3. Self-determination presupposes determining the self to be the cause of an end. 4. Determining your own causality means to “operate as a whole, as something over and above your parts” (Korsgaard 2009a, p. 72). 5. To operate as a whole means exercising one’s will in one’s choices. 7. Since particularistic willing is impossible, willing must be universal. 8. Universal willing means willing your maxims to be universal laws. 9. Hence, to be an agent commits one to the categorical imperative (FUL).

4 The example by which Korsgaard tries to show how the self can be governed by a poor form of autonomy seems inspired Jane Austen’s novel *Emma:* Harriet chooses to be governed by Emma’s will. According to Korsgaard, this is “autonomous action and yet it is *defective* as autonomous action. Harriet is self-governed and yet she is not, for she allows herself to be governed by Emma” (Korsgaard 2009a, p. 162; italics in the original).

5 The following passage from *The Sources of Normativity* highlights the structure of her argument: “The problem faced by the free will is this: the will must have a law, but because the will is free, it must be its own law. And nothing determines what that law must be. *All that it has to be is a law.* Now consider the content of the categorical imperative, as represented by the Formula of Universal Law. The categorical imperative merely tells us to chose a law. Its only constraint on our choice is that it has the form of a law. And nothing determines what the law must be. *All that it has to be is a law.* Therefore the categorical imperative is the law of a free will” (Korsgaard 1996a, p. 98; italics in the original). See also (Korsgaard 1996c, p. 166) where Korsgaard attributes this argument, which rests on a comparison between willing and the content of the categorical imperative, to Kant.

6 I will come back to this point in Section 4 where I outline Korsgaard’s argument in more detail.

7 John Rawls illustrates the point with the following example: “In a game of baseball if a batter were to ask ‚Can I have four strikes?’ it would be assumed that he was asking what the rule was; and if, when told what the rule was, he were to say that he meant that on this occasion he thought it would be best on the whole for him to have four strikes rather than three, this would be most kindly taken as a joke” (Rawls 1955/1999, p. 38).

8 The various formulations of the CI are: Formula of Universal Law (FUL): *“(A)ct only in accordance with that maxim through which you can at the same time can will that it become a universal law*” (Kant 1785/1996, p. 73; G 4:421; italics in the original). Formula of the Law of Nature (FLN): *“(A)ct as if the maxim of your action were to become*

*by your will a* universal law of nature” (Kant 1785/1996, p. 73; G 4:421; italics in the original). Formula of Humanity (FH): “*So act that you use humanity, whether in your own person or in the person of any other, always at the same time as an end, never merely as a means*” (Kant 1785/1996, p. 80; G 4:429; italics in the original). Formula of Autonomy (FA): “(T)he third practical principle of the will” is “the idea *of the will of every rational*

*being as a will giving universal law*” (Kant 1785/1996, p. 81; G 4:431; italics in the original). Formula of the Realm of Ends (FRE): “(A)ct in accordance with the maxims of a member giving universal laws for a merely possible realm of ends” (Kant 1785/1996, p. 88; G 4:439). Note that Mary Gregor’s translation uses the term “kingdom of ends”. However, since “realm of ends” seems more apt as a translation of “Reich der Zwecke” I use it in this paper for all other occurences of Kant’s phrase “kingdom of ends”.

9 Auxter (1982, ch. 5 and ch.8) considers FUL to be paramount. Wood (1999) rejects the identification of the moral law with FUL; he argues “that it is FA rather than FUL which should be considered the ‘universal formula’” and that FA is superior to FH, which again is superior to FUL (Wood 1999, pp. 182–183). Guyer (2005, ch. 7) emphasizes the central role of the Formula of Humanity. O’Neill does not single out one of the formulas as central, but aims to show that the three main formulas of the categorical imperative are equivalent (O’Neill 1989, ch. 7).

10 Apart from the arguments already offered above, there is an additional reason for considering the Formula of a Realm of Ends as crucial: it provides, as I try to show in Pauer-Studer (2016), a link between Kant’s ethics and his philosophy of right.

11 Examples like the following come to one’s mind: discriminating and marginalizing others, subduing and exploiting them, let alone forcing them into slavery or torturing or killing them. According to Allen Wood, FRE demands “the *exclusion* of ends that *in principle* cannot be shared between rational beings (such as those requiring deception or coercion) and the *furthering* of ends that unite people (such as those involving mutual respect and mutual aid)” (Wood 1999, p. 169; italics in the original). As Paul Guyer expresses it, only the promotion of those ends is permissible which can form “a systematic whole of private ends” (Guyer 2007, p. 100).

12 This is worked out more fully in Pauer-Studer (2016, pp. 138–152).

13 This means to examine whether those affected by a maxim do have (or do not have) a normative reason for reasonably rejecting it.

14 For critical discussions of Kant’s tests see O’Neill (1989, ch. 5 and 7); Korsgaard (1996b); Wood (1999, pp. 82–110 and pp. 139–155).

15 The distinction I draw here between two senses of ‘constitutive rule’ makes room for the difference between ‘rule’ and ‘principle’. This is important for answering a possible objection to applying the notions of ‘regulative rule’ and ‘constitutive rule’ to the categorical imperative, namely that the categorical imperative amounts to a principle, but not, strictly speaking, to a rule. The second meaning of ‘constitutive rule’ which I outline above, involves constitutive conditions that then might make up the content of a principle. I thank an anonymous reviewer for suggesting to elucidate this point.

16 My understanding of a transcendental argument is that such an argument consists not merely in the exposition of necessary presuppositions of X (i.e. the conditions of the possibility of X), but that it has a reflexive structure: in the process of laying bare the presuppositions we gain insight why those presuppositions are indispensable and hence why their assumption seems justified - or, as I assumed above, why the principle embracing those presuppositions seems justified. Note that such a reading is more in the spirit of Kant’s exposition of a transcendental argument than a mere reconstruction in terms of necessary presuppositions (which is often taken already to amount to a trancendental argument). I would like to add a qualificiation: Though the insight into the indispensability of the presuppostions definitely adds *support* to their assumption, I do not consider the mere inclusion of a reflexivity condition sufficient for a *full* justification of the ‚conditions of the possibility of’. This is why I add a condition of “reflective approval” which appeals to the normative reasons rational (i.e. reasonable) agents have for agreeing on the principle whose content includes the conditions of the possibility of X, in other words, the constitutive conditions. A detailed discussion of the structure of transcendental arguments is, however, beyond the scope of this paper.

17 An end is conditionally valuable if its worth depends on certain subjective desires and incentives. Such relative ends are, as Kant argues, “only the ground of hypothetical imperatives” (Kant 1785/1996, p. 78; G 4:428).

18 Note that the notion of ‘normative reason’ is open to either a realist or constructivist interpretation or a hybrid form combining a fact-based realist reading of reasons with a constructivist understanding of the practical standpoints from which one’s judgments about reasons are assessed. Within this paper I cannot deal with those questions; suffice it to say that I am inclined to defend a realist underpinning of reasons so that constructivism is not all the way down.

19 This is an application of Kant’s term, which he used for designating a legitimate political order, to the sphere of social morality.

20 Velleman (2009) formulates this point in terms of an objective practical judgment being “unappealable”, meaning that “(s)omeone who arrives at this judgment will consequently find that reconsidering it from another point of view will make no difference, since the judgment will be confirmed from whatever point of view he considers it” (p. 167). Velleman defends an alternative constitutive account of agency, relying on principles such as consistency, coherence and intelligibility - principles that, he claims, tend to drive us towards morality. Velleman’s modest version of constitutivism escapes the bad-action-problem; however, the difficulty is that the connection between the principles of agency and morality (which we consider a main contribution of constitutivism) remains in a way obscure.

21 This step in my account makes room for the Formula of Autonomy (FA). Autonomy in the form of self-legislation secures the individual will’s commitment to the categorical imperative.

22 By drawing the distinction between the moral law and the ethical categorical imperatives Korsgaard also intends to answer the worry that Kant’s ethics is restricted to an isolated first-person perspective and hence to “private reasons”.

23 Pursuing that line, one might criticize that my account falls back on a hypothetical justification of morality. I think the arguments above show that the approach I take does not amount to a hypothetical grounding of the moral law (i.e. the categorical imperative in the form of FRE) in terms of instrumental reasons (particularly not in terms of reasons for maximizing one’s advantage). I thank an anonymous reviewer and also Johan Gersel and Niels de Haan for pressing me on that point.

24 In his discussion of Korsgaard’s account, Douglas Lavin objects that constitutivism (i.e. the “attempt to extract binding principles from the nature of agency”) seems incompatible with the capacity to violate a principle (Lavin 2004, p. 453). This criticism does not touch the position defended here. First of all, the constitutive conditions of morality are not based on the nature of agency; and, second, an agent might not only violate the regulative rules of morality, but also commit himself to a principle that is in tension with the constitutive conditions of morality.

25 In other words, the crucial contribution of Korsgaard’s work lies in elucidating the connections between agency and morality; the part which seems problematic is that in her version of constitutivism the normative standards seem an outcome of agency - a point on which David Enoch’s well-known objections to constitutivism focus (Enoch 2006, 2011). I have tried to show that constitutivism can break away from the hardly defendable assumption that agency per se grounds normativity and morality, and, furthermore, that such a revised form constitutivism provides important insights about the grounding conditions of morality and moral identity. For a detailed discussion and rejection of Enoch’s objections along those lines see Rosati (2016). Rosati sees the main contribution of constitutivism in exploring the relation between agency and morality.

## References

[CR1] Allison HE (1990). Kant’s theory of freedom.

[CR2] Auxter T (1982). Kant’s moral teleology.

[CR3] Cohen GA, Korsgaard CM (1996). Reason, humanity, and the moral law. The sources of normativity.

[CR4] Enoch D (2006). Agency, shmagency: why normativity won’t come from what is constitutive of action. Philos Rev.

[CR5] Enoch D, Brady MS (2011). Shmagency revisited. New waves in metaethics.

[CR6] Guyer P (2005). Kant’s system of nature and freedom: selected essays.

[CR7] Guyer P (2007). Kant’s groundwork for the metaphysics of morals: a reader’s guide.

[CR8] Kant I, Kant I (1785). Groundwork of the metaphysics of morals. Practical philosophy.

[CR9] Kant I, Immanuel K (1793). Religion within the boundaries of mere reason and other writings. Religion and rational theology.

[CR10] Korsgaard CM (1996). The sources of normativity.

[CR11] Korsgaard CM, Korsgaard CM (1996). The right to lie, Kant on dealing with evil. Creating the kingdom of ends.

[CR12] Korsgaard CM, Korsgaard CM (1996). Morality as freedom. Creating the kingdom of ends.

[CR13] Korsgaard CM, Korsgaard CM (1996). Reply. The sources of normativity.

[CR14] Korsgaard CM (2007). Autonomy and the second-person within: a commentary on Stephen Darwall’s *The second-person standpoint*. Ethics.

[CR15] Korsgaard CM (2009). Self-constitution: agency, identity, and integrity.

[CR16] Korsgaard CM (2009). The activity of reason. Proceedings and Addresses of the APA.

[CR17] Lavin D (2004). Practical reason and the possibility of error. Ethics.

[CR18] O’Neill O (1989). Constructions of reason. Explorations of Kant’s practical philosophy.

[CR19] Pauer-Studer H (2016). A community of rational beings’. Kant’s realm of ends and the distinction between internal and external freedom. Kant-Studien.

[CR20] Rawls J (1955/1999) Two concepts of rules. In: Rawls J (1999) Collected papers. Harvard University Press, Cambridge, pp 20–46

[CR21] Rosati CS (2016) Agents and shmagents: an essay on agency and normativity. In: Shafer-Landau R (ed) Oxford studies in metaethics, vol 11. Oxford University Press, Oxford, pp 102–125

[CR22] Searle JR (1995). The construction of social reality.

[CR23] Sidgwick H (1907). The methods of ethics, Seventh edition.

[CR24] Sussman D (2005). Perversity of the heart. Philos Rev.

[CR25] Velleman JD (2009). How we get along.

[CR26] Wallace RJ (2009). The publicity of reasons. Philosophical perspectives. Ethics.

[CR27] Wood AW (1999). Kant’s ethical thought.

